# Rational control of meniscus-guided coating for organic photovoltaics

**DOI:** 10.1126/sciadv.adg9021

**Published:** 2023-08-02

**Authors:** Zhong Zheng, Jianqiu Wang, Junzhen Ren, Shijie Wang, Yafei Wang, Wei Ma, Lei Zheng, Hao Li, Yanjie Tang, Shaoqing Zhang, Jianhui Hou

**Affiliations:** ^1^ School of Chemistry and Biology Engineering, University of Science and Technology Beijing, Beijing 100083, P. R. China.; ^2^ State Key Laboratory of Polymer Physics and Chemistry, Beijing National Laboratory for Molecular Sciences, Institute of Chemistry, Chinese Academy of Sciences, Beijing 100190, P. R. China.; ^3^ University of Chinese Academy of Sciences Beijing 100049, P. R. China.; ^4^ Xi'an Jiaotong University, Xi'an 710049, P. R. China.; ^5^ Shanghai Polytechnic University, Shanghai 201209, P. R. China.

## Abstract

Meniscus-guided coating exhibiting outstanding depositing accuracy, functional diversity, and operating convenience is widely used in printing process of photovoltaic electronics. However, current studies about hydrodynamic behaviors of bulk heterojunction ink are still superficial, and the key dynamic parameter dominating film formation is still not found. Here, we establish the principle of accurately evaluate the Hamaker constant and reveal the critical effect of precursor film length in determining flow evolution, the polymer aggregation, and final morphology. A shorter precursor film is beneficial to restraining chain relaxation, enhancing molecular orientation and mobility. On the basis of our precursor film-length prediction method proposed in this work, the optimal coating speed can be accurately traced. Last, a 18.39% power conversion efficiency has been achieved in 3-cm^2^ cell based on bulk heterojunction fabricated by blade coating, which shows few reduce from 19.40% in a 0.04-cm^2^ cell based on spin coating.

## Teaser

A principle for precursor film length modulating is established to facilitate the preparation of efficient OSC.

## INTRODUCTION

As a focus technology in frontier electronics, organic solar cells (OSCs) with bulk heterojunction (BHJ) emerge as promising photovoltaic devices owing to the high flexibility and solution processability (*1*–*5*). Featured by high ink utilization and easy operation, meniscus-guided coating (MGC) acts as an ideal technology for the large-area solution processing of BHJ (*2*, *3*, *6*–*8*). In BHJ, where the photo-induced charge generation takes place, the segmental configuration and phase separation in donors and acceptors play important roles in determining the photovoltaic performance (*2*, *4*, *9*, *10*). Benefitted from the precise control of morphology, the highest power conversion efficiency (PCE) of single-junction OSC is approaching 20% (*11*, *12*). From laboratory to fabrication, however, hardly can the high PCEs be fully maintained because of the difference between spin coating (SC) and MGC (*2*, *3*, *5*–*8*, *13*–*16*). In SC, the huge inertia in ink produces strong shearing, and the polymer chains can be highly oriented (*13*, *14*, *17*, *18*). Thus, the thermodynamic property of ink is almost the only item to be considered when designing materials. In MGC, on the contrary, the intensities of force components are comparable, so that the detail force balance under meniscus makes the film deposition sensitive to each item affecting the force field (*2*, *3*, *5*–*8*, *15*, *16*, *19*, *20*). The complexities in resolving and analyzing hydrodynamic items make it challenging to establish a valid principle for screening the materials and operating conditions for MGC. This problem leads to the trial and error when performing MGC in OSC. Therefore, revealing the origin for fluid evolution and modeling the solute aggregate in MGC are substantial for OSC and even more broad electronics.

In MGC, the meniscus profile is a joint result of forces in solution bulks and interfaces (*7*, *8*). For example, the shape of macroscopic coating bead is determined by interfacial tension and solution pressure (*7*, *8*, *19*, *20*). For a steady bead on substrate, the famous Young’s equation is used in describing the relationships among interfacial tensions and macroscopic contact angel θ. As the thickness of liquid layer attenuates to micron or even nanometer scale around moving contact line (MCL), the mesoscopic force items increase and stand out as substantial components accounting for the meniscus profile (*21*–*25*). In this case, several mesoscopic forces, such as van Der Waals (VDW) and electrostatic and solvation forces, have been used to correlate the classical Young’s equations and coincident well with experimental results (*26*–*30*). On the basis of the correlations, there is a thin layer of liquid exists at the edge of bead, which is called precursor film (PF) (*21*–*30*). The geometric profile of PF and the flow field distribution inside determine the microcosmic hydrodynamic properties and further the aggregation evolution (*31*). Hence, the fluid zone in PF can be treated as the space with confined force field where mesoscopic properties mould the microcosmic behavior during film formation (*21*, *22*, *28*–*31*). The PF offers us a reliable pathway to approach the force field affecting the morphology and to reveal the key parameters determining the MGC validity with given conditions.

Here, we establish a principle of controlling PF length (*L*_P_) in large-area MGC of BHJ, aiming for transferring the high PCE from laboratory to fabrication. Using multiple BHJ systems into the studies, we reveal the critical effect of *L*_P_ in determining the configuration evolution and the final morphology. By tracing the polymer configuration and aggregation states, the *L*_P_-induced changing in joint VDW forces is demonstrated by the Hamaker constant (*A*). A smaller *L*_P_ is beneficial to restraining the chain relaxation in PF and thus helpful to enhance the molecular orientation. The generality of this argument is proven in MGC performed under various conditions and BHJs. Benefitted from the smaller *L*_P_, restrained relaxation, order molecular orientation, and high mobility, the 3-cm^2^ OSC based on blade-coated poly[1-(5-{4,8-bis[5-(2-ethylhexyl)-4-fluorothiophen-2-yl]-6-methylbenzo[1,2-b:4,5-b′]dithiophen-2-yl}thiophen-2-yl)-5,7-bis(2-ethylhexyl)-3-(5-methylthiophen-2-yl)-4H,8H-benzo[1,2-c:4,5-c′]dithiophene-4,8-dione] (PBDB-TF):BTP-eC9 BHJ exhibits 17.11% PCE, which is the highest value among the PCEs achieved from OSCs with the same area and BHJ. Moreover, we reveal the interrelationship connecting *L*_P_, optimal coating speed, optimal BHJ thickness, and intrinsic ink parameters. These interrelationship offer us an opportunity to rationally optimize *L*_P_ by tuning the initial coating conditions. The method has been used in predicting coating conditions of large-area ternary BHJ (PBDB-TF:FTCC-Br:BTP-eC9, showing 19.40% PCE in 0.04-cm^2^ SC-processed cell), and a 18.39% PCE is obtained in 3-cm^2^ OSC. We expect that our *L*_P_ modeling in MGC can provide a reliable guidance for further BHJ printing and material design.

## RESULTS

### Basic description of MGC and the hydrodynamic origin of PF

**Fig. 1.**
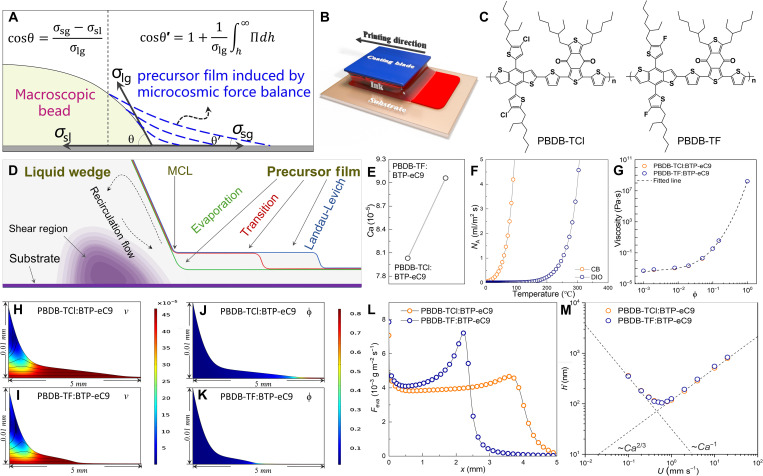
The illustration of PF in the MGC of BHJ materials in organic photovoltaics. (**A**) Sketch of liquid layer thinning at the edge of bead. From macroscopic to microcosmic situation, the VDW forces become non-ignorable. The macroscopic contact angle θ is replaced by microcosmic angle θ′. The Young's equation should be corrected by the disjoining pressure (σ_sg_, σ_sl_, and σ_lg_ are solid-gas, solid-liquid, and liquid-gas interfacial tensions, respectively). (**B**) Sketch of blade coating. (**C**) Molecular structures of PBDB-TF and PBDB-TCl. (**D**) Sketch of meniscus at the edge of the coating bead. Three primary fluid regimes are listed. The left part beside stagnation point (SP) is liquid wedge, while the right part is PF. (**E**) Capillary number (*Ca*) of the inks based on PBDB-TF:BTP-eC9 and PBDB-TCl:BTP-eC9 (the conditions of inks are listed in Materials and Methods). (**F**) Rates of volatilization of chlorobenzene (CB) and 1,8-diiooctane (DIO), calculated by Fick's law. (**G**) Relationship between ink viscosity and polymer volume fraction (ϕ). (**H**) Fluid simulation of streamlines in the bead of PBDB-TF:BTP-eC9. (**I**) Fluid simulation of streamlines in the bead of PBDB-TCl:BTP-eC9. (**J**) Fluid simulation of ϕ in the bead of PBDB-TF:BTP-eC9. (**K**) Fluid simulation of ϕ in the bead of PBDB-TCl:BTP-eC9. (**L**) Rates of solvent volatilization obtained from simulation. The *x* is the position referring to the start point of simulation. (**M**) Experimental and simulated thicknesses (*h*′) of the films coated from the two inks. The dash line is the fitting curves. MCL, moving contact line.

For a steady bead on substrate, the famous Young's equation cosθ = (σ_sg_ − σ_sl_)/σ_lg_ is used in describing the relationships among interfacial tensions and macroscopic contact angel θ (*28*, *29*, *32*–*34*). At the edge of bead, the triple-phase contact line (TCL) would not suddenly changed to solid/gas phase line mathematically, which demonstrates the necessity of the correction including mesoscopic forces. Several works confirm the validity of the correction of $\cos\theta^{\prime }=1+(1/\sigma_{\mathrm{lg}})\int_{h}^{\infty }\text{Π}(h)dh$ (*35*, *36*), where θ′ is the microcosmic contact angel, *h* is thickness of meniscus, and Π(*h*) is the disjoining pressure (Fig. 1A). As expressed in the corrected equation, the Π(*h*) represents the thinning rate of liquid film at the edge of bead. Coincident with the theoretical prediction, there is indeed a PF that exists at the edge of bead. As an item representing the summed VDW attractions within the fluid zones located between meniscus and substrate, the Hamaker constant (*A*) shows close correlation between Π(*h*), *L*_P_, and PF thickness (*h*_0_) (*27*, *31*). In the MGC of conjugated polymer ink, the critical effect of *L*_P_ can be indicated by *A* (*26*, *31*, *37*).

The sketch of MGC apparatuses (here in this work is blade coating) is shown in Fig. 1B. Detail experimental conditions can be found in Materials and Methods. The structures of conjugated polymers, which contribute major attractions to rheological properties, are shown in Fig. 1C. According to references (*38*, *39*), the two polymers exhibit almost the same energy levels and absorption spectra, with the only difference in chemical structure exists in the side chain. The difference in halogen substituent causes different inter/intramolecular actions.

In MGC, several characteristic flows such as Marangoni recirculation, shearing flow, and laminar flow exist in liquid wedge, near MCL, and in PF, respectively (Fig. 1D) (*8*, *19*). The MCL locates at the position SP where the direction of streamline is vertical to the meniscus profile (*40*). Depending on *L*_P_, the fluid distribution can be classified into three fluid regimes (Fig. 1D) (*7*). We first get the parameters to be used in simulation, such as capillary number (*Ca*) (Fig. 1E), volatilization flux (*N*_A_), (Fig. 1F), and viscosity (μ) (Fig. 1G). Then, the profiles, streamlines, velocities, shearing rate, and solute volume fractions in fluid fields are simulated (Fig. 1, H to J). Detail parameters for simulation are listed in Materials and Methods. The velocities in both the inks vary markedly in liquid wedges but stable in PFs. On the basis of the streamlines, the apparent laminar flows are present in PFs. Figure 1 (J and K) shows that the increasing of ϕ mainly happen at the ends of PFs, which is in accord with the position (*x*)–dependent rate of volatilizing (*F*_eva_) shown in Fig. 1l. Except the issues above, the *L*_P_ of PBDB-TCl:BTP-eC9 is obviously longer than the *L*_P_ of PBDB-TF:BTP-eC9. On the basis of the coating speed (*U*)–dependent *h* tested from various films (Fig. 1M), we can infer that both the MGCs of PBDB-TCl: and PBDB-TF:BTP-eC9 in our work locate belong to transition regime (*7*).

### The description of AS1, AS2, and AS3

**Fig. 2.**
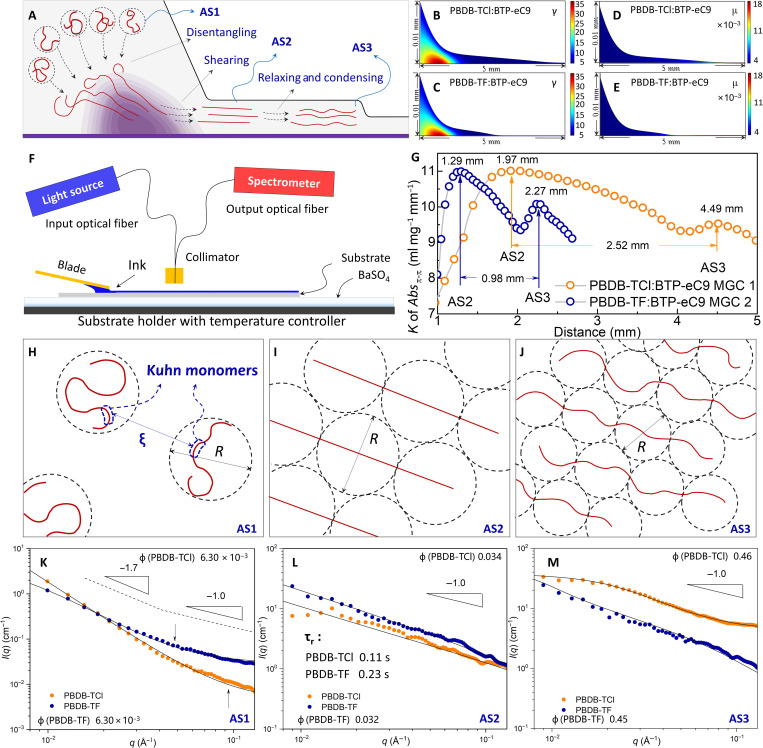
The characterizations for the states of AS1, AS2, and AS3. (**A**) Sketch of aggregate states and the characteristic point in meniscus. (**B** and **C**) Distributions of shearing rates under meniscus in PBDB-TF:BTP-eC9 and PBDB-TCl:BTP-eC9, respectively. (**D** and **E**) Distributions of μ under meniscus in PBDB-TF:BTP-eC9 and PBDB-TCl:BTP-eC9, respectively. (**F**) Diagram of in situ fast response absorption spectra (ISFR-Abs) characterization. (**G**) ISFR-Abs spectra evolution of PBDB-TCl:BTP-eC9 PF and PBDB-TF:BTP-eC9 PF (the corresponding conditions are MGC 1 and MGC 2 shown in Materials and Methods, respectively). The *K* of *Abs*_π-π_ is the absorption coefficient of the absorption around 610 nm caused by π-π stacking in polymer. The sketches of polymer configurations at (H), AS1, (I) AS2, and (J) AS3, respectively. In (**H**), the circular dash lines represent the pervaded volumes of polymer chains. ξ is the correlation length, *R* is the diameter of pervaded volume, and ɛ is the intensity coefficient of interaction. The ɛ is only dependent on the type of particles and independent of the volume fraction. In (**I**) and (**J**), the circular dash lines represent the volumes of Kuhn monomers. The ξ_h_ is the hydrodynamic screening length of Kuhn monomer. The small-angle x-ray scattering (SAXS) data of the two inks at (**K**) AS1, (**L**), AS2, and (**M**) AS3.

The characteristic states are illustrated in Fig. 2A. AS1 refers to the PBDB-TF aggregation state (AS) in pristine ink. When passing through the shear region, the segments of polymer become aligned and oriented (this state is AS2) (*7*, *8*, *19*). The existences of shear regions are proven by the apparent shearing rate variations near the MCL in Fig. 2 (B and C). In PF, the segmental relaxation takes place until the point of intense solvent volatilization (AS3, the position of *F*_eva_ maximum appears). At AS3, the μ increases markedly (Fig. 2, D and E).

Among AS1, AS2, and AS3, the position of AS2 cannot be obtained directly from the simulation. We carry out ISFR-Abs characterization (illustrated in Fig. 2F) to find out the position of AS2. By using a spectrometer with 20-ms resolution, we can resolve the spectral evolution from liquid wedge to PF. Because the thickness of the ink in liquid wedge is high, which exceeds the valid range of Beer-Lambert law, we only collect the data in PF. As shown in Fig. 2G, there are two peaks in each *K* of *Abs*_π-π_. The first peak is higher than the second one, which indicates a more ordered packing in the first state. According to our description of MGC that the relaxation restrains the close packing in AS2 and causes the lower *K* at AS3, the behaviors of the two peaks coincide well with AS2 and AS3, respectively. By equaling the position (product of time and *U*) of AS3 in ISFR-Abs to the position in COMSOL, we can achieve the position of AS2 by calculating the difference between the two peaks in Fig. 2G.

At AS1 of semidilute solution, the polymer chains are surrounded by solvent molecules. We design a set of instruments (fig. S1) to make sure the position exposing at x-ray can be finely tuned. On the basis of the characteristic positions provided by COMSOL simulation and ISFR-Abs, the information of small-angle x-ray scattering (SAXS) at any given point can be obtained. We carry out SAXS characterization on pristine inks (AS1) using CB as solvent (Fig. 2I). In low-*q* region, the asymptotic power law of *I*(*q*) ~ *q*^ν^ (ν = −1.7) demonstrates the worm-like global configuration (*41*–*43*); in high-*q* region, the asymptotic power law of *I*(*q*) ~ *q*^ν^ (ν = −1) demonstrates the rod-like local configuration (Fig. 2F) (*41*–*43*). This observation supports the using of flexible cylinder model for AS1 (*41*–*44*). The crossover (*q*_c_) from the rod-like to coil behavior can be obtained from the *q*, where the form factor ν switching from −1.7 to 1 (*41*–*43*). The *q*_c_ of PBDB-TCl:BTP-eC9 and PBDB-TF:BTP-eC9 inks at AS1 is 0.10 and 0.06 Å^−1^, respectively. According to the relationship between *q*_c_ and Kuhn length (*b*), 2*b* ≥ 3.5/*q*_c_ (*41*–*43*), we can obtain the minimum of *b* (18 and 29 Å for PBDB-TCl:BTP-eC9 and PBDB-TF:BTP-eC9, respectively), which are in accord with the values achieved in the fitting based on flexible cylinder model (see Table 1).

**Table 1.** The results of SAXS fitting at AS1, AS2, and AS3.

Inks	AS1				AS2				AS3			
	*q*_c_ (Å^−1^)	*b* (Å)	ξ (Å)	*R* (Å)	*b* (Å)	ξ_h_ (Å)	*R* (Å)	*L* (Å)	*b* (Å)	ξ_h_ (Å)	*R* (Å)	*L* (Å)
PBDB-TCl:BTP-eC9	0.10	20	1400	127	1108	1.4 × 10^4^	737	998	16	23	49	999
PBDB-TF:BTP-eC9	0.06	35	3263	187	1360	1.8 × 10^4^	847	1000	346	495	353	1000

Getting through shearing region, the polymer chains become highly aligned at AS2 (Fig. 2G) (*7*, *8*, *19*). The hypothesis is verified by the SAXS data in Fig. 2J. The form factor ν is steady on about −1 across the whole detecting range, and 2*b* (the persistence length) is even higher than contour length (*L*). Those are clear indications that both the local and global chains exhibit roughly rod-like configuration at AS2, in which the configuration of polymer chain is highly governed by the chain adjacent (*41*–*43*). At this stage, segments can still move only if the size of segment is lower than ($\xi_{h}=b\phi_{\mathrm{AS}2}^{-0.45}$) (*45*, *46*). In the length scale larger than ξ_h_, the hydrodynamic interaction is screened by surrounding chains. Hence, from AS2 on, we can treat the ink as a large aggregate consisting of closely packed Kuhn monomers and the solvent in pervaded volumes. The *b* can be obtained in the SAXS data by using flexible cylinder model. All the corresponding parameters are shown in Table 1.

From AS2 on, the polymer chains step into the stage in laminar flows without shearing. According to our hypothesis sketched in Fig. 2A, the polymer relaxation would take place with the characteristic relaxation time of monomer in semidilute solution, τ_r_ = 6πμ*N*^0.588^*b*^3^/*kT* (*45*, *47*), where *N* is the degree of polymerization. Calculating the τ_r_ with μ extracted from Figs. 1G and 2, D and E, *N* extracted from figs. S2 to S5 and table S1, the τ_r_ of PBDB-TCl (0.36 s) at AS2 is much shorter than PBDB-TF (0.58 s). It means that the configuration of PBDB-TF at AS3 is more rigid than PBDB-TCl (Fig. 2H). This deduction is also proven by *b* and τ_r_ obtained from SAXS data at AS3 (Fig. 2K). At AS3, the τ_r_ of PBDB-TCl (2.59 × 10^−6^ s) at AS3 is much shorter than PBDB-TF (2.36 × 10^−2^ s). The decreased but higher *b* of PBDB-TF than PBDB-TCl reveals the influence of *L*_P_ on chain configuration.

**Fig. 3.**
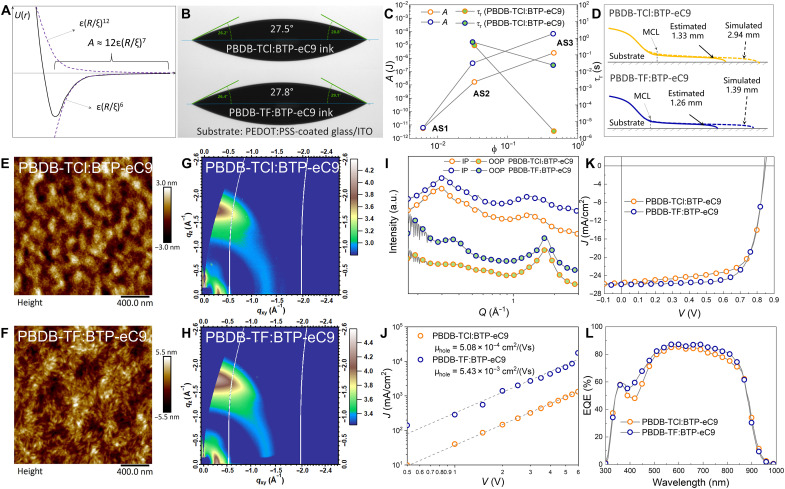
Mesoscopic origin of BHJ morphology evolution and the corresponding influence on photovoltaic performance. (**A**) Diagram of the effective interaction potential *U*(ξ) versus the distance between Kuhn monomers (ξ). Within the distance larger than ξ_c_, the attraction is much greater than repulsion. (**B**) Contact angles of the two inks on substrate. (**C**) Calculated parameters at AS1-AS3. (**D**) Comparisons of *L*_P_ obtained from COMSOL simulation and experimental data. The atomic force microscope (AFM) height images of the films processed from (**E**) PBDB-TCl:BTP-eC9 and (**F**) PBDB-TF:BTP-eC9–based inks. The grazing incidence wide angle x-ray scattering (GIWAXS) data of the films processed from (**G**) PBDB-TCl:BTP-eC9 and (**H**) PBDB-TF:BTP-eC9–based inks. (**I**) In-plane (IP) and out-of-plane (OOP) profiles of the GIWAXS patterns in (G) and (H). (**J**) Space charge–limited current (SCLC) mobility of the OSCs with BHJs based on PBDB-TF:BTP-eC9 and PBDB-TCl:BTP-eC9. (**K**) Current density–voltage (*J-V*) curves of 3-cm^2^ OSCs based on PBDB-TF:BTP-eC9 and PBDB-TCl:BTP-eC9 BHJs under the illumination of AM 1.5 G (100 mW/cm^2^). (**L**) External quantum efficiency (EQE) of 3-cm^2^ OSCs based on PBDB-TF:BTP-eC9 and PBDB-TCl:BTP-eC9 BHJs under the illumination of AM 1.5 G (100 mW/cm^2^). a.u., arbitrary units.

Furthermore, we establish the calculation method of *A* at AS1, AS2, and AS3. As illustrated in Fig. 2F, the statistical distance between Kuhn monomers is ξ and the effective interaction potential is *U*(ξ). A quantitative relationship has been established elsewhere that *A* = −12ξ*U*(ξ)/*R* (Fig. 3A) (*31*–*45*). In the region of ξ > ξ_c_ [the critical distance where *U*(ξ) is minimized], the attraction has the primary contribution in *U*(ξ). At AS1, the aggregate state of dilute solution fits the situation well. The *A* can be expressed by

$$A\approx 12\varepsilon {\left(R/\xi \right)}^{7}$$ (1)

At SP, several researches report the relationship of *A* = 6πσ_lg_*h*^2^ (*31*, *44*, *45*, *48*, *49*). The σ_lg_ can be obtained by the pendant drop method shown in fig. S6 and table S2, and the spreading coefficient (*S*) can be achieved by σ_lg_ and contact angle (Fig. 3B).Thus, the values of the ɛ of PBDB-TCl:BTP-eC9 and PBDB-TF:BTP-eC9 are 8.66 × 10^−6^ and 2.48 × 10^−4^ J, respectively. The *R* can be obtained by *R* = *b*(*v*/*b*^3^)^0.18^*N*^0.588^ (*45*). The *v* is excluded volume, and the corresponding characterization method has been listed in Materials and Methods. At AS1, *v* can be estimated as 1.45 and 2.73 nm^3^ for PBDB-TCl:BTP-eC9 and PBDB-TF:BTP-eC9, respectively. The ξ in dilute or semidilute solution can be expressed as ξ = *b*(*b*^3^/*v*)^0.23^ϕ^−0.77^ (*45*, *46*). The values of the parameters at AS1 are shown in Table 1.

At AS2 and AS3, the ξ in Eq. 1 should be replaced by ξ_h_

$$A=1.25\varepsilon \phi_{P}^{1.925}$$ (2)

With all the parameters achieved, we plot Fig. 3C and summarize the values in Table 1. As seen, the *A* becomes larger from AS1 to AS3, in both the cases of PBDB-TCl:BTP-eC9 and PBDB-TF:BTP-eC9. The *A* of PBDB-TCl:BTP-eC9 at each state is lower than that of PBDB-TF:BTP-eC9. That is the evidence of the stronger interaction in PBDB-TF–based ink.

### The influence of *L*_P_ on morphology of BHJ

The difference in *L*_P_, which acts as the critical factor for MGC of BHJ, leads to the emerging of different *A* at AS1, AS2, and AS3. In this study, the *L*_P_ can be obtained from COMSOL simulation. Moreover, we establish another convenient method to estimate the *L*_P_. Referring to literatures, the *L*_P_ of nonvolatile ink can be evaluated by the parameters at AS1: $L_{P}=(1/Ca){\left(SA/6\pi \sigma_{\mathrm{lg}}^{2}\right)}^{0.5}$ (*37*), where *S* is spreading coefficient which equals to σ_lg_ (1 – cosθ). The *A* at AS1 equals to 12πμ*U*σ_lg_/ρ*g*. Considering evaporation and assuming that the solvent is completely removed at the end of PF, we correct the equation of *L*_P_ by

$$L_{P}=2\phi_{0}\sigma_{\mathrm{lg}}{\left[\frac{2(1-\cos\theta )}{\mu U\rho g}\right]}^{\frac{1}{2}}$$ (3)

where ϕ_0_ is initial volume fraction of polymer. By using Eq. 3, the estimated *L*_P_ of PBDB-TCl:BTP-eC9 (1.33 mm) and PBDB-TF:BTP-eC9 (1.26 mm) is obtained (Fig. 3D). Although there is a large difference from the simulated values (2.94 and 1.39 mm), the sort is the same. It means that we can use Eq. 3 to estimate *L*_P_.

Forming from larger *L*_P_ and more intense relaxation, the PBDB-TCl chains exhibit apparent coil-like configuration, which is verified by the atomic force microscopy (AFM) height images of the deposits (Fig. 3, E and F). The fibrous texture of PBDB-TF:BTP-eC9 is clear and distinguishable, while the fibrous texture of PBDB-TCl:BTP-eC9 is fuzzy. The fiber in PBDB-TF:BTP-eC9 is more stout and aligned compared to PBDB-TCl:BTP-eC9, that is because of the lower *L*_P_ and thus the weak chain relaxation in PBDB-TF:BTP-eC9.

The more order orientation is also detected in grazing incidence wide-angle x-ray scattering (GIWAXS) patterns. All samples are prepared by vacuum pumping the coated films for 30 min. As shown in Fig. 3 (G, H, and I), PBDB-TCl:BTP-eC9 and PBDB-TF:BTP-eC9 exhibit very similar scattering profiles with preferential face-on packing and similar peak location for laminar (100) and π-π (010) stacking. The (100) reflections of PBDB-TCl:BTP-eC9 and PBDB-TF:BTP-eC9 locate at *q*_xy_ = 0.31 and 0.29 Å^−1^ in in-plane (IP) direction, which correspond to *d*-spacing of 20 and 22 Å, respectively. The (010) reflections of PBDB-TCl:BTP-eC9 and PBDB-TF:BTP-eC9 locate at *q*_z_ = 1.73 and 1.74 Å^−1^ in out-of-plane (OOP) direction, which correspond to *d*-spacing of 3 Å, respectively. Both the scatterings corresponding to (100) and (010) reflections are more pronounced in PBDB-TF:BTP-eC9, demonstrating the more ordered packing induced by the restrained relaxation in PF. This orientation is often beneficial for efficient charge transport and collection out of the device. The more ordered morphology of PBDB-TF:BTP-eC9 contributes to the hole mobility (μ_hole_). The μ_hole_ of PBDB-TF:BTP-eC9 and PBDB-TCl:BTP-eC9 is measured using space charge–limited current (SCLC) method (Fig. 3J). The μ_hole_ of PBDB-TCl:BTP-eC9 is 5.08 × 10^−4^ cm^2^ V^−1^ s^−1^, which is much lower than PBDB-TF:BTP-eC9 (5.43 × 10^−3^ cm^2^ V^−1^ s^−1^). The high mobility of PBDB-TF:BTP-eC9 is consistent with the carrier lifetime (τ_carrier_). Excited with a white light-emitting diode (LED), the transient photovoltage (TPV) of photovoltaic devices are collected (fig. S7). On the basis of single exponential fitting, the τ_carrier_ of devices with PBDB-TCl:BTP-eC9 and PBDB-TF:BTP-eC9 BHJs are 97.40 and 168.71 ns, respectively. Therefore, the lower *L*_P_ is beneficial to enhancing the molecular orientation and thus the mobility.

Using the MGC of PBDB-TCl:BTP-eC9 and PBDB-TF:BTP-eC9 in practical fabrication, two 3-cm^2^ OSCs are prepared. Here, we compare the PBDB-TCl:BTP-eC9 [0.6 mm/s; polymer concentration of 11 mg/ml in CB and 0.5% DIO, donor/acceptor ratio of 1:1 weight % (wt %), and polymer molecular weight (*M*_w_) of 59,819 g/mol] and PBDB-TF:BTP-eC9 (0.6 mm/s; polymer concentration of 11 mg/ml in CB and 0.5% DIO, donor/acceptor ratio of 1:1 wt %, and polymer *M*_w_ of 50,672 g/mol), respectively. As the BHJ exhibits different morphologies and mobility, the performance of OSCs are obviously different. As shown in Fig. 3K and Table 2, the PBDB-TF:BTP-eC9– and PBDB-TCl:BTP-eC9–based OSCs show similar open circuit voltages (*V*_oc_) but different short circuit current densities (*J*_sc_) and fill factors (FFs). The FF of PBDB-TCl:BTP-eC9–based OSC is 71.25%, which is lower than the FF of PBDB-TF:BTP-eC9–based OSC (73.11%). The *J*_sc_ of PBDB-TCl:BTP-eC9–based OSC is 25.63 mA/cm^2^, which is slightly lower than the *J*_sc_ of PBDB-TF:BTP-eC9–based OSC (25.99 mA/cm^2^). As the two *J*_sc_ are verified by the *J*_sc_ calculated by external quantum efficiency (EQE) data in Fig. 3l (the calculated *J*_sc_ are 25.20 and 26.25 mA/cm^2^ respectively, and both the deviations are lower than 2%), the differences in *J*_sc_ and FF can be attributed to the different morphology and mobility. As a result, the PCE of PBDB-TF:BTP-eC9–based 3 cm^2^ OSC is as high as 16.15%, which is higher than the 15.34% PCE of PBDB-TCl:BTP-eC9–based OSC. Compared to the PCEs obtained from OSCs processed by SC, the PCE of PBDB-TF:BTP-eC9–based OSC can be well maintained in 3-cm^2^ MGC owing to the lower *L*_P_.

**Table 2.** The results of estimated *L*_P_ and photovoltaic parameters of OSCs under AM 1.5 G (100 mW cm^−2^). The statistical results are obtained from five OSCs.

BHJ	Condition	*L*_P_ (cm)*	*h*′_opt_ (nm)	*U* (mm/s)	Area (cm × cm)	*V*_oc_ (V)	*J*_sc_ (mA cm^−2^)	FF (%)	PCE (%)
PBDB-TCl:BTP-eC9	SC	–	101	–	0.2 × 0.2	0.85	26.87	77.50	17.70 (17.55 ± 0.19)
	MGC 1	1.33	105	0.6	1 × 3	0.84	25.63	71.25	15.34 (15.12 ± 0.11)
PBDB-TF:BTP-eC9	SC	–	103	–	0.2 × 0.2	0.85	27.01	77.57	17.81 (17.65 ± 0.14)
	MGC 1	1.21	101	0.4	1 × 3	0.84	26.55	75.87	16.92 (16.70 ± 0.20)
	MGC 2	1.26	105	0.6	1 × 3	0.85	25.99	73.11	16.15 (15.98 ± 0.08)
	MGC 3	0.60	102	2.2	1 × 3	0.85	26.13	77.04	17.11 (16.92 ± 0.06)
	MGC 4	0.79	210	1.2	1 × 3	0.84	26.85	63.40	14.30 (14.13 ± 0.12)
	MGC 5	2.53	30	0.2	1 × 3	0.54	11.12	36.97	2.22 (1.83 ± 0.32)
PTB7-Th:BTP-eC9	SC	–	106	–	0.2 × 0.2	0.68	26.06	71.22	12.62 (12.42 ± 0.10)
	MGC 1	1.28	92	0.4	1 × 3	0.67	25.82	67.69	11.71 (11.56 ± 0.12)
	MGC 2	1.25	100	0.6	1 × 3	0.68	24.88	66.32	11.22 (10.96 ± 0.21)
	MGC 3	0.62	100	1.8	1 × 3	0.68	24.83	66.39	11.21 (10.94 ± 0.25)
	MGC 4	0.22	700	20.0	1 × 3	0.65	24.11	52.52	8.23 (7.84 ± 0.32)
	MGC 5	0.47	60	2.4	1 × 3	0.67	16.28	53.63	5.85 (4.79 ± 1.03)
PTVT-T:BTP-eC9	SC	–	102	–	0.2 × 0.2	0.78	26.87	77.49	16.24 (16.01 ± 0.16)
	MGC 1	0.88	94	0.4	1 × 3	0.78	26.58	76.06	15.77 (15.33 ± 0.29)
	MGC 2	0.89	100	1.6	1 × 3	0.78	25.32	75.75	14.96 (14.63 ± 0.28)
	MGC 3	0.53	105	4.8	1 × 3	0.77	26.83	77.35	15.98 (15.72 ± 0.23)
	MGC 4	0.40	423	4.0	1 × 3	0.74	27.87	68.90	14.21 (13.97 ± 0.20)
	MGC 5	2.51	46	1.0	1 × 3	0.74	21.62	63.94	10.23 (10.02 ± 0.17)
PBDB-TCl:FTCC-Br:BTP-eC9 (1:0.4:0.8)	SC	–	109	–	0.2 × 0.2	0.885	26.83	79.93	18.98 (18.76 ± 0.21)
	MGC	0.14	111	0.4	1 × 3	0.880	26.49	76.06	17.73 (17.51 ± 0.18)
PBDB-TF:FTCC-Br:BTP-eC9 (1:0.4:0.8)	SC	–	122	–	0.2 × 0.2	0.885	26.91	81.46	19.40 (19.25 ± 0.13)
	MGC	0.13	126	0.4	1 × 3	0.880	26.47	78.95	18.39 (18.01 ± 0.36)

*The *L*_P_ is estimated by Eq. 3.

### The accurate prediction of *L*_P_ and optimal *U*

**Fig. 4. F4:**
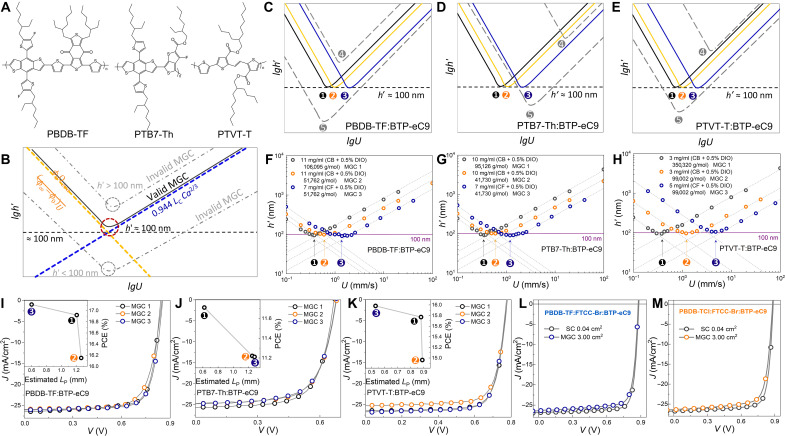
The key function of *L*_P_ and the prediction of *U*_opt_. (**A**) Molecular structures of the polymers taken into universality study about *L*_P_. (**B**) Diagram of mapping of (*lgh*′)/(*lgU*). In MGC, when *U* is low, the *h*′ obeys *U*^−1^ (orange dash line and equation), and when *U* is high, the *h*′ obeys *U*^2/3^ (blue dash line and equation). Considering that 100 nm is the typical optimal thickness of most BHJ, only if the intersection point of the two lines locates around 100 nm (black fold line) can the 100-nm-thick film be produced from transition regime. If the intersection point is much higher than 100 nm, never can the 100-nm-thick BHJ be produced; if the intersection point is much lower than 100 nm, then the 100-nm-thick BHJ can only be produced from evaporation regime or Landau-Levich regime, those are harmful to morphology. *L*_c_ is capillary length [*L*_c_ = (σ_lg_/*ρg*)^1/2^], ϕ_c_ is the infinite ϕ (equals to 1), and *Q*_eva_ is the evaporation flux per unit of length. (**C**) Diagrams of fold lines corresponding to five conditions of PBDB-TF:BTP-eC9 inks. (D) Diagrams of fold lines corresponding to five conditions of PTB7-Th:BTP-eC9 inks. (**E**) Diagrams of fold lines corresponding to five conditions of PTVT-T:BTP-eC9 inks. The detail of conditions are shown in Materials and Methods. (**F**) Measured *U*-dependent *h*′ plots of three valid conditions of PBDB-TF:BTP-eC9 inks. (**G**) Measured *U*-dependent *h*′ plots of three valid conditions of PTB7-Th:BTP-eC9 inks. (**H**) Measured *U*-dependent *h*′ plots of three valid conditions of PTVT-T:BTP-eC9 inks. The detail of conditions are shown in legends. The *J-V* curves of OSCs based on (**I**) PBDB-TF:BTP-eC9, (**J**) PTB7-Th:BTP-eC9, and (**K**) PTVT-T:BTP-eC9 BHJs under the illumination of AM 1.5 G (100 mW/cm^2^). The relationships between *L*_P_ and PCEs are shown in inserts. (**L**) *J-V* curves of OSCs based on PBDB-TF:FTCC-Br:BTP-eC9. (**M**) *J-V* curves of OSCs based on PBDB-TCl:FTCC-Br:BTP-eC9.

The universality of *L*_P_ effect can be tested in broad classes of inks with different concentrations, solvents, and conjugated polymers with different backbones. Here, we use three BHJ systems to finish the test (Fig. 4A). Before testing, one thing should be noticed that the comparison of *L*_P_-dependent PCE should be performed in OSCs with 100-nm-thick BHJs, and the polymers in BHJs have the same backbones. To make sure it produces 100-nm-thick BHJ from the transition regime, we need to keep the intersection point of characteristic lines for evaporation and Landau-Levich regimes at around 100 nm. Those conditions with higher or lower intersection points are invalid (Fig. 4B) (*50*). Then, we test the universality in three BHJ systems with 15 condition combinations (Fig. 4, C to E, and Table 2). On the basis of the mapping in Fig. 4B, some inks cannot be used to produce 100-nm-thick BHJ across the whole *lgU* range, and thus, the cells show poor photovoltaic performance (shown in figs. S8 to S10).

Because of the much lower or higher *h*′ than 100 nm, the conditions of MGC 4 and MGC 5 in each BHJ systems are excluded from the comparison of *L*_P_. On the basis of the conditions selected in Fig. 4 (F to H), the estimated *L*_P_ (calculated by Eq. 3) and the corresponding PCEs are characterized. As shown in Fig. 4 (I to K) and Table 2, in each BHJ systems, the lower *L*_P_ produces the higher PCE. As the *L*_P_ is getting shorter, the difference of PCE between MGC and SC decreases. These results provide solid evidence for the universal effect of *L*_P_ in the MGC of OSC. By using the MGC with a 0.60-mm *L*_P_, the PCE of OSC produced from MGC 3 condition is as high as 17.11%, which is the highest value among MGC-processed 3-cm^2^ OSCs. The PCE is similar to the one of 0.04-cm^2^ SC-processed cell (fig. S11).

Moreover, the optimal thickness of deposited BHJ based on given conditions (*h*′_opt_) and the corresponding *L*_P_ can be easily predicted for a given ink. The *h*′_opt_ can be obtained by the intersection of characteristic lines for evaporation and Landau-Levich regimes (*50*–*54*)

$$h_{\mathrm{opt}}^{\prime }={\left(\frac{0.045}{{\text{Δ}}_{\mathrm{ext}}{\text{Δ}}_{\mathrm{eva}}}\right)}^{\frac{3}{5}}$$ (4)

where ${\text{Δ}}_{\mathrm{ext}}={\left(\rho g\right)}^{1/2}{\left(\sigma_{\mathrm{lg}}\right)}^{1/6}{\left(\mu \right)}^{2/3}$ and ${\text{Δ}}_{\mathrm{eva}}={\left[(1-\phi_{0})/(\phi_{0}Q_{\mathrm{eva}})\right]}^{2/3}$. The optimal *U* for the *h*′_opt_ can be achieved by

$$U_{\mathrm{opt}}={\left(\frac{h_{\mathrm{opt}}^{\prime }{\text{Δ}}_{\mathrm{ext}}}{0.045}\right)}^{\frac{3}{2}}$$ (5)

On the basis of *U*_opt_, as well as the ρ, μ, θ, and σ_lg_, the *L*_P_ can be predicted using Eq. 3, so that whether the recipe of ink is suitable to preparing 100-nm BHJ and producing highly efficient OSC can be known without fabricating the device. The effectiveness of this method has been demonstrated by the estimated *L*_P_, *h*′_opt_, and PCE in Table 2. Furthermore, we use this method to obtain *U*_opt_, *h*′_opt_, and the estimated *L*_P_ of two ternary BHJs (PBDB-TCl:FTCC-Br:BTP-eC9 and PBDB-TF:FTCC-Br:BTP-eC9). FTCC-Br is a middle bandgap acceptor (molecular structure of FTCC-Br is shown in fig. S12), which has been successfully used as the third components in highly efficient ternary OSC (*55*). The best PCEs of 0.04-cm^2^ OSCs based on the two ternary BHJs are obtained, those are 18.98% for PBDB-TCl:FTCC-Br:BTP-eC9 and 19.40% for PBDB-TF:FTCC-Br:BTP-eC9, respectively. Then, we use the basic parameters of the two ternary inks (shown in Materials and Methods) to get the *U*_opt_, *h*′_opt_, and the estimated *L*_P_ in MGC. For PBDB-TCl:FTCC-Br:BTP-eC9, those are 0.4 mm/s, 111 nm, and 0.14 cm, respectively; for PBDB-TF:FTCC-Br:BTP-eC9, those are 0.4 mm/s, 126 nm, and 0.13 cm, respectively. By using *U*_opt_ in MGC, two top-ranking PCEs have been achieved in 3-cm^2^ cells. Similar to binary BHJs, as the estimated *L*_P_ of PBDB-TF:FTCC-Br:BTP-eC9–based MGC is shorter than that in PBDB-TCl:FTCC-Br:BTP-eC9–based MGC, the PCEs of the two OSCs fabricated by MGC show obvious difference. For PBDB-TCl:FTCC-Br:BTP-eC9, the PCE is 17.73%; for PBDB-TF:FTCC-Br:BTP-eC9, the PCE is 18.39%. Both the results are the highest values in OSCs as large as 3 cm^2^. The deviations of *J*_sc_ obtained from current density–voltage (*J-V*) curve and EQE spectra are all lower than 2% (fig. S13 and table S3). These results illustrate the accuracy of the relationship between *L*_P_ and PCE and demonstrate the effectiveness of our method in predicting the coating conditions.

## DISCUSSION

In this work, we establish the *L*_P_-modulating principle aiming for applying current BHJ materials in large-area MGC by rational guidance instead of trial and error. Using two similar polymeric donors PBDB-TCl and PBDB-TF into the studies, we reveal the critical effect of *L*_P_ in aggregation evolution under meniscus and clarify the fluid origin of BHJ morphology formation in OSC. By tracing the chain configuration evolution, the Hamaker constants in the confined force field in PF are accurately characterized and the correlation with *L*_P_ is demonstrated. On the basis of these results, we find that a shorter *L*_P_ is beneficial to restraining the chain relaxation and thus helpful to enhance the molecular orientation, for the MGC flows belonging to transition and Landau-Levich regimes. The generality of this argument is proven in MGC that performed under various conditions and BHJs. As most MGC are carried out in transition and Landau-Levich regimes, our finding works in broad BHJ systems and thus can be used for judging the feasibility of MGC. Benefitted from the shorter PF, restrained relaxation, order molecular orientation, and high mobility, the 3-cm^2^ OSC based on blade-coated PBDB-TF:BTP-eC9 BHJ exhibits 17.11% PCE, which is the highest value among the PCEs achieved from binary OSCs with the same area. Moreover, the interrelationship connecting *L*_P_, optimal coating speed, optimal BHJ thickness, and intrinsic ink parameters have been revealed. By using the interrelationship, we achieve the optimized *L*_P_ and *U*_opt_ of two ternary BHJ inks, those show abilities to output 19.40 and 18.98% PCEs in SC-processed OSCs. Last, a 18.39% PCE is obtained in 3-cm^2^ OSC (BHJ is PBDB-TF:FTCC-Br:BTP-eC9). We expect our *L*_P_ modeling in MGC can provide a reliable guidance for further BHJ printing and material design.

## MATERIALS AND METHODS

### Materials

PDINN, PBDB-TF, PBDB-TCl, PTB7-Th, PTVT-T, and BTP-eC9 were purchased from Solarmer Material Inc. Poly(3,4-ethylenedioxythiophene)-poly(styrenesulfonate) (PEDOT:PSS) (CLEVIOS P VP AI 4083) was purchased from Heraeus Inc. All these solvents used in the experiment were commercially available from Acros. The glass/indium tin oxide (ITO) substrates were purchased from Huanan Xiangcheng Inc. The silicon wafer–consisted coating head is purchased from Beijing Moderation Technology Co. LTD.

### Characterization

#### Viscosity

Viscosities were determined using an Ubbelohde viscometer with a viscometer constant of 0.002787 at 25°C, and the viscometer constant was calibrated with 1,2-ethanediol. For measurements conducted at 25°C, the viscometer was immersed in a temperature-controlled water bath. Solutions were allowed to equilibrate at the preset temperature for 15 min before analysis, and viscosity values were averaged over five runs.

#### Surface characterizations

The contact angle, surface tension, and surface free energy were performed on DSA100, Kruss.

#### Molecular weight

The *M*_w_ of the polymer was measured by the Gel Permeation Chromatography (GPC) method with polystyrene as the standard and 1,2,4-tricholorobenzene as the solvent at 160°C using Agilent Technologies PL-GPC220.

#### ISFR-abs

In situ fast response ultraviolet (UV) visible absorption spectroscopy measurements were performed by the OCEAN-FX-VIS-NIR-ES spectrometer. The HL-2000-FHSA light source is purchased from Ocean Optics Inc. The spectrometer using the absorbance mode with the time resolution of 20 ms. The detector collects the absorbance spectra ranged from 400 to 1050 nm during coating. The instruments of ISFR-Abs experiment is displayed in fig. S14. There is a collimator in the “Y” shape optical fiber delivering incidence light and collecting reflecting light. The horizon position of the collimator can be tuned by a microcalliper, and the vertical position is fixed at 60 mm to substrate. The 60-mm distance is confirmed by a charge-coupled device (CCD), which can image the light spot. When the light spot meets minimum, the focal distance is 60 mm.

#### SAXS

Solution sample preparation for SAXS is carried out as the following procedures. The material for substrate in SAXS testing is polyimide (PI) with thickness of 25 μm. The holders are made by stainless steel. The PI substrate produces weak influence on SAXS testing. Before blade coating, we rub 7 μl of initial ink on a starting line on PI substrate with pipette and quickly hold the trace initial ink by the silicon-coating head. The trace initial inks are stable between the substrate and coating head for its large viscosity and low mass. In this experiment, the gap between silicon coating head and substrate is 0.01 mm. During testing, the coating head is held by both blade and substrate. Thus the up limit of additional pressure (Δ*P*) is 2σ_lg_/*L* (*L* is 0.01 mm), which equals to 1.02 × 10^4^ pa. The bared liquid area between the blade and substrate is about 0.01 mm^2^. Thus, the up limit of additional drag force is 1.02 × 10^−4^ N. The sum of 1.02 × 10^−4^ and 2.21 × 10^−5^ N is enough to tightly hold the bead with a gravity of 7.61 × 10^−5^ N, which coincides well with what we observed during the experiment. Referring to each concerned state such as AS1, AS2, and AS3, the distance between blade edge and detecting point is approached by using the micrometers shown in fig. S1. The carrier of substrate can smoothly move on the guideway drove by stepper motor. According to COMSOL simulation, the distances of AS1, AS2, and AS3 can be traced in each ink. SAXS datasets were performed in BL10U1 beam line in Shanghai Synchrotron Radiation Facility. The x-ray energy is 10 keV; the collection time for SAXS is 10 ms; the flux of x-ray photon is 10^13^ counts/s; the detector is PILATUS 2M; the distance between the detector and sample is 27.6 m. At temperature intervals of 25°C, SAXS data are collected. Line collimation is used for incident x-rays on deposited films. Scattered x-rays can be recorded. Intensity of scattering, *I*(*q*), is obtained as a function of *q*, where *q* is considered as 4πsinθ_x_/λ (θ_x_ is the incident angle of x-ray). The SAXS samples are representative of the inks at the various stages. First, the relaxation times for the samples are long enough. The relaxation time of segments (τ_segment_) can be obtained by $\tau_{\mathrm{segment}}=\frac{fb^{2}}{6\pi^{2}kT}{\left(\frac{N}{p}\right)}^{2}$, where *f* and *b* are friction coefficient and Kuhn length, respectively. The *f* of conjugated polymer locates in range of 0.001 to 0.002. In polymer dynamics model, the relaxations are described by *N* different relaxation modes, which is numbered by mode index *p* = 1, 2, 3, …, *N*. According to the equation, the relaxation time of polymers at AS2 is around 7 hours, respectively. Second, the position of the meniscus exposed on x-ray can be finely tuned by the micrometers. The classical flexible cylinder model that is appropriate to describe the conjugated polymers with large π-plane is used for fitting the SAXS data (*42*, *44*). The equation of model is derived and corrected by Chen *et al. *(**48**). The detail is provided in (*48*). The SasView software integrates flexible cylinder model, sphere model, ellipsoid model, and other common used models, which is widely used in SAXS data analyzing (*49*). In this study, we use flexible cylinder model in SasView software to fit the SAXS data.

#### GIWAXS

GIWAXS measurements were performed at beamline 7.3.3 at the Advanced Light Source at Lawrence Berkeley National Laboratory. Samples were prepared on Si substrates using identical blend solutions as those used in devices. The 10-keV x-ray beam was incident at a grazing angle of 0.12° or 0.13°, which maximized the scattering intensity from the samples. The scattered x-rays were detected using a DECTRIS PILATUS 2M photon counting detector. AFM height and phase images were recorded on a Nanoscope V AFM microscope (Bruker), where the tapping mode was used.

#### TPV measurement

The excitation light is white LED. The photovoltage is collected when the LED is shut down. The τ_carrier_ is obtained by single exponential fitting.

#### J-V curves

The *J-V* characterizations were carried out on a KEITHLEY 2400 Precision Source/Measure unit. The illumination of AM 1.5 G (100 mW/cm^2^) was achieved by a XES-70S1 solar simulator (SAN-EI Electric Co. Ltd., AAA grade, photo beam size of 70 mm by 70 mm). A single-crystal Si diode (20 mm by 20 mm) was used to calibrate the irradiation power of the simulator.

#### EQE

The EQE was measured by Solar Cell Spectral Response Measurement System QE-R3011 (Enlitech, Taiwan). The light intensity at each wavelength was calibrated with a single-crystal Si diode.

#### Film thickness

The film thickness was obtained via a surface profilometer (DektakXT, Bruker).

#### Mobolity

The hole mobility was measured by the SCLC method, with the device architecture of glass/ITO/PEDOT:PSS/sample/Au. The equation used for fitting is *J* = ɛ_0_ɛ_r_μ_e_9*V*^2^/8*L*^3^.

#### Preparation of PEDOT:PSS emulsion

The purchased emulsion of PEDOT:PSS (CLEVIOS P VP AI 4083) should be diluted by water with twice the volume of stock solution at room temperature and stirred for 15 min before use.

#### Preparation of PDINN solution

The PDINN (1.0 mg) was dissolved in 1 ml of methanol at room temperature under vigorous stirring in N_2_ glove box.

#### Preparation of BHJ solutions[Fig F4]

For PBDB-TCl:BTP-eC9 SC, the polymer concentration is 8 mg/ml in CF and 0.5% by volume DIO, the donor/acceptor ratio is 1:1 wt %, and the *M*_w_ of polymer is 59,819 g/mol. For PBDB-TCl:BTP-eC9 MGC 1, the polymer concentration is 11 mg/ml in CB and 0.5% by volume DIO, the donor/acceptor ratio is 1:1 wt %, the *M*_w_ of polymer is 59,819 g/mol, ρ is 1110 kg/m^3^, σ_lg_ is 25.91 mN/m, μ is 0.00208 Pa·s, θ is 27.7°, and ϕ_0_ is 0.0063. For PBDB-TF:BTP-eC9 SC, the polymer concentration is 8 mg/ml in CF and 0.5% by volume DIO, the donor/acceptor ratio is 1:1 wt %, and the *M*_w_ of polymer is 50,672 g/mol. For PBDB-TF:BTP-eC9 MGC 1, the polymer concentration is 11 mg/ml in CB and 0.5% by volume DIO, the donor/acceptor ratio is 1:1 wt %, the *M*_w_ of polymer is 106,095 g/mol, ρ is 1110 kg/m^3^, σ_lg_ is 25.58 mN/m, μ is 0.00353 pas, θ is 27.3°, and ϕ_0_ is 0.0061. For PBDB-TF:BTP-eC9 MGC 2, the polymer concentration is 11 mg/ml in CB and 0.5% by volume DIO, the donor/acceptor ratio is 1:1 wt %, the *M*_w_ of polymer is 50,672 g/mol, ρ is 1110 kg/m^3^, σ_lg_ is 25.62 mN/m, μ is 0.00232 pas, θ is 27.8°, and ϕ_0_ is 0.0063. For PBDB-TF:BTP-eC9 MGC 3, the polymer concentration is 7 mg/ml in CF and 0.5% by volume DIO, the donor/acceptor ratio is 1:1 wt %, the *M*_w_ of polymer is 50,672 g/mol, ρ is 1450 kg/m^3^, σ_lg_ is 21.28 mN/m, μ is 0.000749 pas, θ is 23.2°, and ϕ_0_ is 0.0054. For PBDB-TF:BTP-eC9 MGC 4, the polymer concentration is 15 mg/ml in CB and 0.5% by volume DIO, the donor/acceptor ratio is 1:1 wt %, the *M*_w_ of polymer is 50,672 g/mol, ρ is 1100 kg/m^3^, σ_lg_ is 25.86 mN/m, μ is 0.00356 pas, θ is 28.0°, and ϕ_0_ is 0.0068. For PBDB-TF:BTP-eC9 MGC 5, the polymer concentration is 3 mg/ml in CB and 0.5% by volume DIO, the donor/acceptor ratio is 1:1 wt %, the *M*_w_ of polymer is 50,672 g/mol, ρ is 1110 kg/m^3^, σ_lg_ is 26.78 mN/m, μ is 0.00117 pas, θ is 28.2°, and ϕ_0_ is 0.0049. For PTB7-Th:BTP-eC9 SC, the polymer concentration is 8 mg/ml in CF and 0.5% by volume DIO, the donor/acceptor ratio is 1:1 wt %, and the *M*_w_ of polymer is 41,730 g/mol.

PTB7-Th:BTP-eC9 MGC 1, the polymer concentration is 10 mg/ml in CB and 0.5% by volume DIO, the donor/acceptor ratio is 1:1 wt %, the *M*_w_ of polymer is 95,126 g/mol, ρ is 1110 kg/m^3^, σ_lg_ is 25.61 mN/m, μ is 0.00328 pas, θ is 27.8°, and ϕ_0_ is 0.0062. For PTB7-Th:BTP-eC9 MGC 2, the polymer concentration is 10 mg/ml in CB and 0.5% by volume DIO, the donor/acceptor ratio is 1:1 wt %, the *M*_w_ of polymer is 41,730 g/mol, ρ is 1110 kg/m^3^, σ_lg_ is 25.48 mN/m, μ is 0.00235 pas, θ is 27.5°, and ϕ_0_ is 0.0064. For PTB7-Th:BTP-eC9 MGC 3, the polymer concentration is 7 mg/ml in CF and 0.5% by volume DIO, the donor/acceptor ratio is 1:1 wt %, the *M*_w_ of polymer is 41,730 g/mol, ρ is 1450 kg/m^3^, σ_lg_ is 21.87 mN/m, μ is 0.000836 pas, θ is 23.1°, and ϕ_0_ is 0.0052. For PTB7-Th:BTP-eC9 MGC 4, the polymer concentration is 4 mg/ml in CB and 0.5% by volume DIO, the donor/acceptor ratio is 1:1 wt %, the *M*_w_ of polymer is 41,730 g/mol, ρ is 1100 kg/m^3^, σ_lg_ is 25.95 mN/m, μ is 0.0013 pas, θ is 27.9°, and ϕ_0_ is 0.0046. For PTB7-Th:BTP-eC9 MGC 5, the polymer concentration is 1 mg/ml in CB and 0.5% by volume DIO, the donor/acceptor ratio is 1:1 wt %, the *M*_w_ of polymer is 41,730 g/mol, ρ is 1100 kg/m^3^, σ_lg_ is 25.62 mN/m, μ is 0.000272 pas, θ is 27.8°, and ϕ_0_ is 0.0016. For PTVT-T:BTP-eC9 SC, the polymer concentration is 5 mg/ml in CF and 0.5% by volume DIO, the donor/acceptor ratio is 1:1 wt %, and the *M*_w_ of polymer is 99,002 g/mol. For PTVT-T:BTP-eC9 MGC 1, the polymer concentration is 3 mg/ml in CB and 0.5% by volume DIO, the donor/acceptor ratio is 1:1 wt%, the *M*_w_ of polymer is 350,320 g/mol, ρ is 1110 kg/m^3^, σ_lg_ is 25.3 mN/m, μ is 0.00338 pas, θ is 27.2°, and ϕ_0_ is 0.0045. For PTVT-T:BTP-eC9 MGC 2, the polymer concentration is 3 mg/ml in CB and 0.5% by volume DIO, the donor/acceptor ratio is 1:1 wt %, the *M*_w_ of polymer is 99,002 g/mol, ρ is 1100 kg/m^3^, σ_lg_ is 25.36 mN/m, μ is 0.000874 pas, θ is 27.1°, and ϕ_0_ is 0.0046. For PTVT-T:BTP-eC9 MGC 3, the polymer concentration is 5 mg/ml in CF and 0.5% by volume DIO, the donor/acceptor ratio is 1:1 wt %, the *M*_w_ of polymer is 99,002 g/mol, ρ is 1470 kg/m^3^, σ_lg_ is 21.65 mN/m, μ is 0.000375 pas, θ is 22.8°, and ϕ_0_ is 0.0050. For PTVT-T:BTP-eC9 MGC 4, the polymer concentration is 11 mg/ml in CB and 0.5% by volume DIO, the donor/acceptor ratio is 1:1 wt %, the *M*_w_ of polymer is 99,002 g/mol, ρ is 1100 kg/m^3^, σ_lg_ is 25.38 mN/m, μ is 0.00304 pas, θ is 27.2°, and ϕ_0_ is 0.0061. For PTVT-T:BTP-eC9 MGC 5, the polymer concentration is 13 mg/ml in CB and 0.5% by volume DIO, the donor/acceptor ratio is 1:1 wt %, the *M*_w_ of polymer is 99,002 g/mol, ρ is 1100 kg/m^3^, σ_lg_ is 25.35 mN/m, μ is 0.000436 pas, θ is 27.3°, and ϕ_0_ is 0.0072. For PBDB-TCl:FTCC-Br:BTP-eC9 SC, the polymer concentration is 10 mg/ml in CB and 0.2% by volume DIO, the donor/acceptor ratio is 1:0.4:0.8 wt %, and the *M*_w_ of polymer is 59,819 g/mol. For PBDB-TCl:FTCC-Br:BTP-eC9 MGC, the polymer concentration is 10 mg/ml in CB and 0.2% by volume DIO, the donor/acceptor ratio is 1:0.4:0.8 wt %, the *M*_w_ of polymer is 59,819 g/mol, and ϕ_0_ is 7.40 × 10^−3^, ρ is 1250 kg/m^3^, σ_lg_ is 25.88 mN/m, μ is 0.0046 pas, *Q*_eva_ is 5.78 × 10^−9^ m^2^/s, and θ is 31.0°. For PBDB-TF:FTCC-Br:BTP-eC9 SC, the polymer concentration is 15 mg/ml in CB and 0.2% by volume DIO, the donor/acceptor ratio is 1:0.4:0.8 wt %, the *M*_w_ of polymer is 50,672 g/mol. For PBDB-TF:FTCC-Br:BTP-eC9 MGC, the polymer concentration is 15 mg/ml in CB and 0.2% by volume DIO, the donor/acceptor ratio is 1:0.4:0.8 wt %, the *M*_w_ of polymer is 50,672 g/mol, and ϕ_0_ is 7.40 × 10^−3^, ρ is 1250 kg/m^3^, σ_lg_ is 25.78 mN/m, μ is 0.00487 pas, *Q*_eva_ is 7.52 × 10^−9^ m^2^/s, and θ is 28.0°.

#### The blade coating operation

The blade in this work is mono-crystalline silicon wafer with a thickness of 0.2 mm. The wafer should be tightly attached on the slider with an angle of 15°. A micrometer is used to tune the gap between wafer and substrate to 0.01 mm. The coating head is a silicon wafer treated by fine polishing. The silicon wafer is composed of single-crystal silicon that only a pronounced peak corresponding to (400) crystal face exists on x-ray diffraction pattern. The roughness of the wafer is 0.2 nm across the entire surface. No pattern and fine structure are processed on the surface. The surface energy of the wafer is 39.82 mN/m. The wafer could be directly used without UV ozone treatment. The slider is drove by a stepping motor with an operating limit faster than 0.01 mm/s. The temperature of supporting platform for substrate should be controlled. For PEDOT:PSS blade coating, 10 mm/s and 50°C are used. For BHJ blade coating, 25°C are used. The coating speeds should be tuned to designed values. For PDINN blade coating, 20 mm/s and 25 centigrade are used.

#### The fabrication of OSC

Glass/ITO substrates were cleaned with detergent, deionized (DI) water, acetone, and isopropanol. After 15-min UV ozone, the ITO substrates were cooled down for further using. For SC cells, the emulsion of PEDOT:PSS (CLEVIOS P VP AI 4083) was spin-coated on top of ITO substrates with 4000 rpm; for MGC cells, the emulsion of PEDOT:PSS (CLEVIOS P VP AI 4083) was blade-coated on top of ITO substrates, followed by 10 min annealing at 150°C. After annealing, the PEDOT:PSS-coated ITO substrates were transferred into N_2_ glove box. Then, the solutions of BHJ were spin-coated or blade-coated on the basis of the used conditions. All the BHJs (PBDB-TCl:BTP-eC9, PBDB-TF:BTP-eC9, PTB7-Th:BTP-eC9, PTVT-T:BTP-eC9, PBDB-TCl:FTCC-Br:BTP-eC9, and PBDB-TF:FTCC-Br:BTP-eC9) should be annealed at 100°C and 10 min. After thermal annealing, for SC cells, the PDINN layer was spin-coated with 3000 rpm; for MGC cells, the PDINN layer was blade-coated at 25 centigrade and 30 mm/s. After that the 200 nm of Ag was thermally evaporated under high vacuum (ca. 3 × 10^−4^ Pa). The cell area was 0.04 (SC) or 3 cm^2^ (MGC), which is defined by apertures.

#### The determining of Ca and N_A_

In low Reynolds numbers (*Re*) MGC, the fluid regime and, hence, the quality of the final film are dominated by *Ca* of the bead. We measure σ_lg_ and μ of inks to calculate *Ca* by μ*U*/σ_lg_. The *N*_A_ can be achieved according to Fick’s law, $N_{A}=\frac{Dp(p_{A1}-p_{A2})}{R_{g\mathrm{as}}TL_{g}p_{\mathrm{Bm}}}$, where *R*_gas_ is ideal gas constant, *D* is evaporation coefficient, *p*_A1_ and *p*_A2_ are partial pressure of chlorobenzene, *L*_g_ is thickness of gas layer, *p* is total pressure of gas layer, *p*_Bm_ is the logarithmic mean of 1,8-diiodooctane partial pressure of the stationary gas layer, and *p*/*p*_Bm_ is drift factor.

#### COMSOL simulation

Numerical simulation was performed using a commercial finite element software package COMSOL Multiphysics. The laminar two-phase flow, moving mesh interface, and transport of diluted species interface are used to model the coating process. The simulation is time dependent. We let the run time of the simulation to be long enough to obtain a stationary result. The dynamics of fluid is defined by Navier-Stokes equations

$$\rho \frac{\partial U}{\partial t}+\rho (U\;\cdot \nabla )U=\nabla \;\cdot \{-pI+\mu [\nabla U+{\left(\nabla U\right)}^{T}]\}+\rho g$$

∇⋅U=0

where ρ is density, *U* is velocity, *p* is pressure, *g* is the gravitational acceleration, μ is the viscosity of water, and *I* is identity matrix. In addition, the COMSOL built-in moving mesh method is implanted to describe the shape change of the fluid interface and the effect of surface tension and mass flux. The diffusion-convection equation is used to calculate the solute concentration, *c*, in the fluid

$$\frac{\partial c}{\partial t}-D_{1}\nabla^{2}c+U\nabla c=0$$

where *D*_1_ is the diffusion coefficient of the solute. The evaporation of the solvent is described by the follow diffusion equation,

$$\frac{\partial c_{v}}{\partial t}-D_{2}\nabla^{2}c_{v}=0$$

where *D*_2_ is the diffusion coefficient and *c*_v_ is the vapor concentration in air. For the fluid flow, gravity is considered. A pulling velocity, *U*, is given at the boundary (1), and boundary (3) and boundary (5) are open boundaries. Boundary (2) is the interface where the surface tension, sigma1, is set and a mass flux, *M*_f_, is set. The mass flux presents the fluid loss by the evaporation. For the vapor diffusion in the air, the vapor concentration at boundary (4) is set to be zero. An inward flux of vapor, *J*_0_, is set at boundary (2)

$$J_{0}={N_{A0}}^{*}[{c_{b}}^{*}(1-\phi )-c_{v}]$$

where *c*_b_ is saturated vapor concentration, ϕ is volume fraction of solute, and *N*_A0_ is an evaporation rate constant. In addition, ϕ is defined by

$$\phi =c/{\rho_{2}}^{*}\;M_{2}$$

where ρ_2_ and *M*_2_ are density and relative molecular mass of solute, respectively. Then, the mass flux, *M*_f_, can be defined as

$$M_{f}={J_{0}}^{*}M_{1}$$

where *M*_1_ is the elative molecular mass of solvent. For the solute concentration in the fluid, boundary (3) is set to be a contest value, *c*_0_, and an inward flux, *J*_1_, is set at boundary (2)

$$J_{1}=c^{*}\;M_{1}/{\rho_{1}}^{*}J_{0}$$

where ρ_1_ and *M*_1_ are density and relative molecular mass of solvent, respectively. The dynamic viscosity of the fluid is dependent on the solute concentration. The relationship between dynamic viscosity and solute concentration is measured by experiments. The evaporation rate of solvent is measured by experiments, and the value of *D*_2_ is set to fit the experimental evaporation rate.

### Determination of polymer excluded volume

The scale of excluded volume can be evaluated by the product of Kuhn length, *d*-spacing of π-π stacking in OOP direction, and *d*-spacing of (100) stacking in IP direction. According to Fig. 3 (G to I), for PBDB-TCl, the *d*-spacing of π-π stacking in OOP direction is 3.63 Å and *d*-spacing of (100) stacking in IP direction is 20.47 Å. For PBDB-TF, the *d*-spacing of π-π stacking in OOP direction is 3.60 Å and *d*-spacing of (100) stacking in IP direction is 21.67 Å. The Kuhn lengths of polymers at different ASs can be found in Table 1.
