# Supplementary material for: Rational control of meniscus-guided coating for organic photovoltaics
Source: Sci Adv. 2023 Aug 2;9(31):eadg9021. doi: 10.1126/sciadv.adg9021 (PMC10396288; doi:10.1126/sciadv.adg9021)
Supplement: Supplementary file 1 — Figs. S1 to S15 Tables S1 to S3 [file sciadv.adg9021_sm.pdf]

Science Advances
AAAS

Supplementary Materials for

Rational control of meniscus-guided coating for organic photovoltaics

Zhong Zheng *et al.*

Corresponding author: Zhong Zheng, zhongzheng@ustb.edu.cn; Jianhui Hou, hjhzlz@iccas.ac.cn

Sci. Adv. **9**, eadg9021 (2023)
DOI: 10.1126/sciadv.adg9021

This PDF file includes:

Figs. S1 to S15
Tables S1 to S3

Supplementary Section 1. Apparatuses of SAXS characterization.

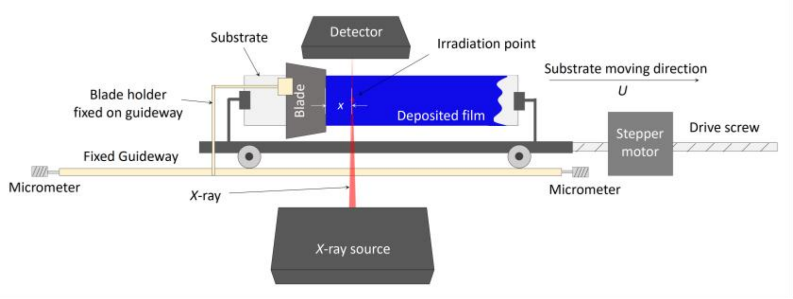

Figure S1 | The apparatuses of SAXS characterization in this work. The distance between the blade edge and irradiation point (*x*) can be finely tuned by the micrometers. The direction of optical pathway is normal to the plane of substrate.

Supplementary Section 2. Molecular weight characterization of the polymers.

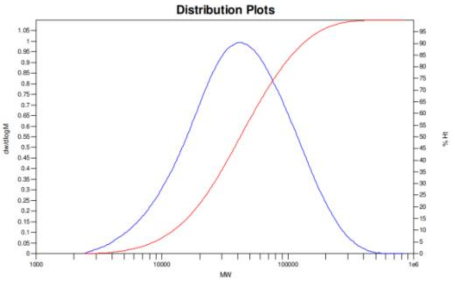

Figure S2 | The molecular weight data of PBDB-TCl polymers in this study based on gel

permeation chromatography method (GPC).

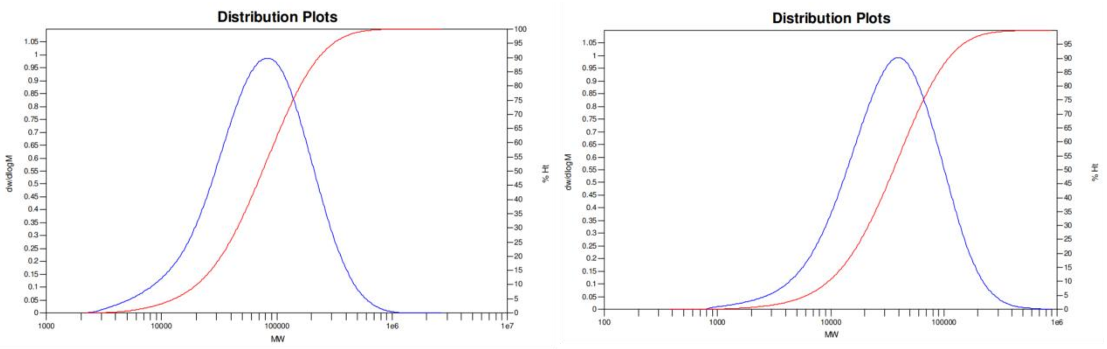

Figure S3 | The molecular weight data of PBDB-TF polymers in this study based on GPC.

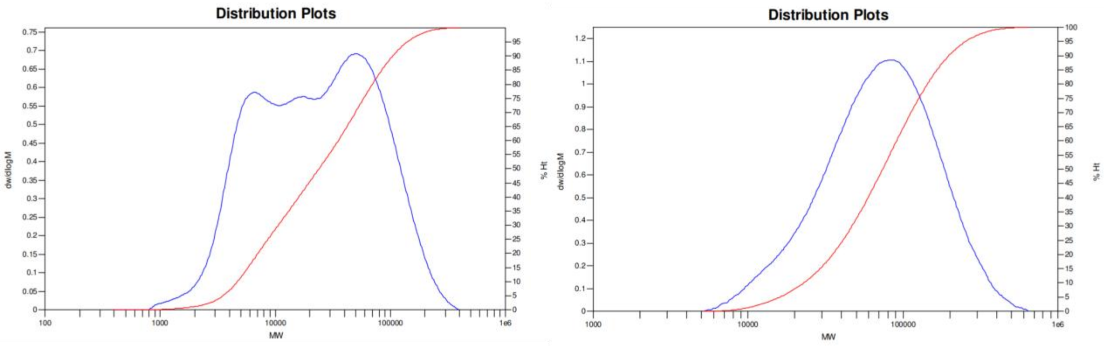

Figure S4 | The molecular weight data of PTB7-Th polymers in this study based on GPC.

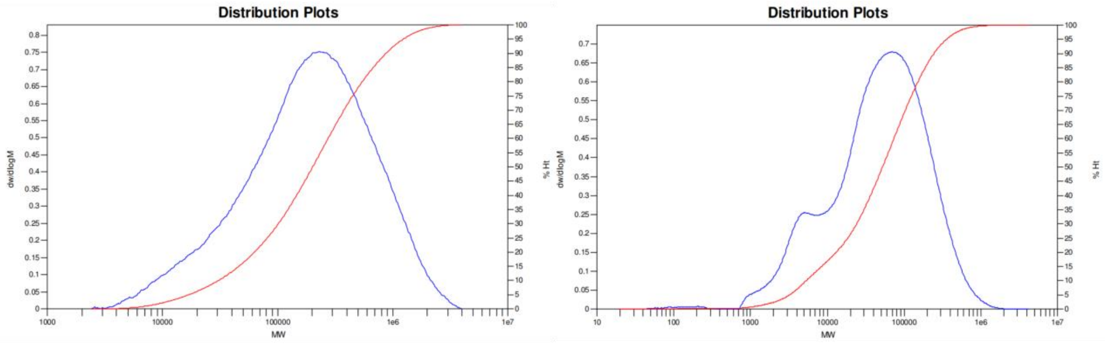

Figure S5 | The molecular weight data of PTVT-T polymers in this study based on GPC.

Table S1 | Corresponding Mw from Figure S1 to S4.

Polymers	M_w (g/mol)
PBDB-TCl	59819
PBDB-TF	106095
PBDB-TF	50672
PTB7-Th	95126
PTB7-Th	41730
PTVT-T	350320
PTVT-T	99002

Supplementary Section 3. The characterization of σ_{lg} of PBDB-TCl and PBDB-TF.

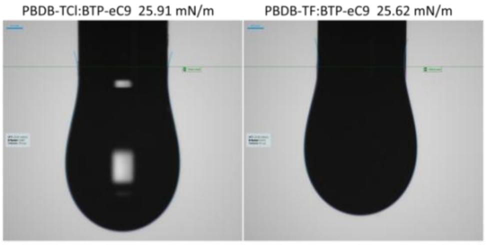

Figure S6 | The σ_{lg} of PBDB-TCl:BTP-eC9 and PBDB-TF:BTP-eC9 inks measured by pendant drop method at 25 centigrade.

Table S2 | The σ_{lg} of PBDB-TCl:BTP-eC9 and PBDB-TF:BTP-eC9 inks at 25 centigrade.

Inks	σ_{lg}
PBDB-TCl:BTP-eC9 (M_w 59819)	25.91
PBDB-TF:BTP-eC9 (M_w 50672)	25.62

Supplementary Section 4. The carrier lifetimes of devices with PBDB-TCl:BTP-eC9 and PBDB-TF:BTP-eC9 BHJs, respectively.

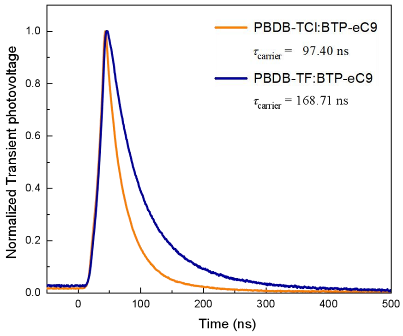

Figure S7 The data of transient photovoltage experiment. The excitation light is white light emitting diode (LED). The photovoltage is collected when the LED is shut down. The carrier lifetime ($\tau_{carrier}$) is obtained by single exponential fitting.

Supplementary Section 5. The Supplementary photovoltaic performance.

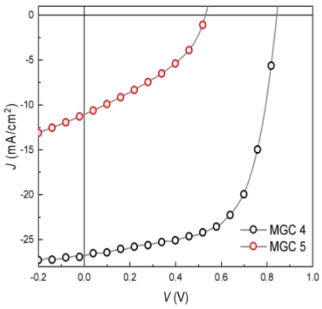

Figure S8 | The current density-voltage J-V curves of 3 cm^2 OSCs with PEDOT:PSS/BHJ/PDINN

layers fabricated by blade coating. The testing is carried out under the illumination of AM 1.5G 100 mW/cm^2. The BHJ materials are PBDB-TF:BTP-eC9. The MGC conditions used for BHJ are MGC 4 and 5.

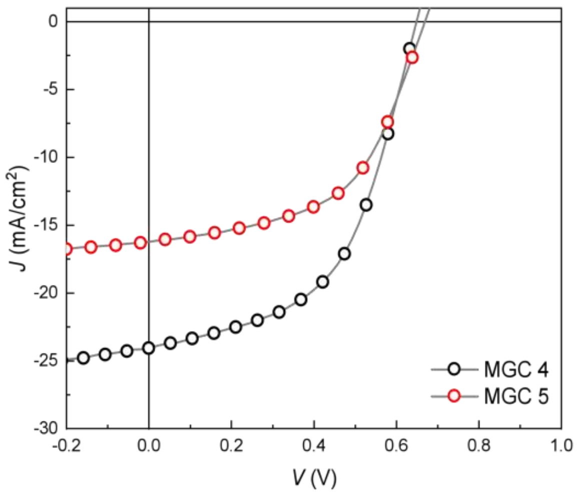

Figure S9 | The current density-voltage *J-V* curves of 3 cm^2 OSCs with PEDOT:PSS/BHJ/PDINN layers fabricated by blade coating. The testing is carried out under the illumination of AM 1.5G 100 mW/cm^2. The BHJ materials are PTB7-Th:BTP-eC9. The MGC conditions used for BHJ are MGC 4 and 5.

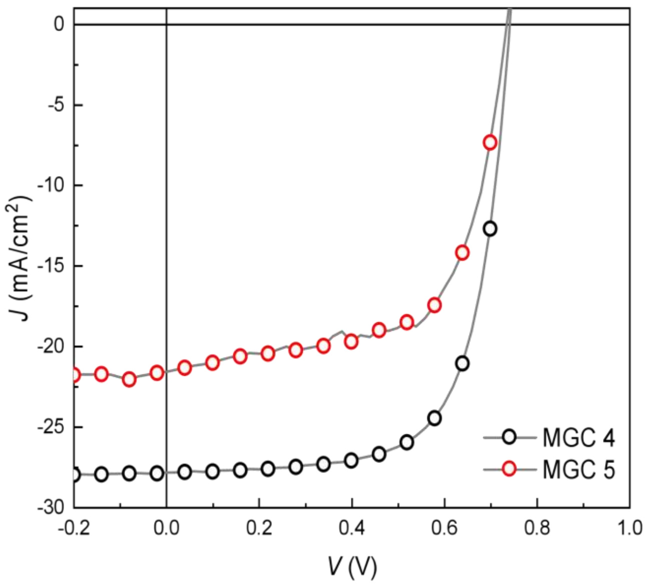

Figure S10 | The current density-voltage *J-V* curves of 3 cm^2 OSCs with PEDOT:PSS/BHJ/PDINN layers fabricated by blade coating. The testing is carried out under the illumination of AM 1.5G 100 mW/cm^2. The BHJ materials are PTVT-T:BTP-eC9. The MGC conditions used for BHJ are MGC 4 and 5.

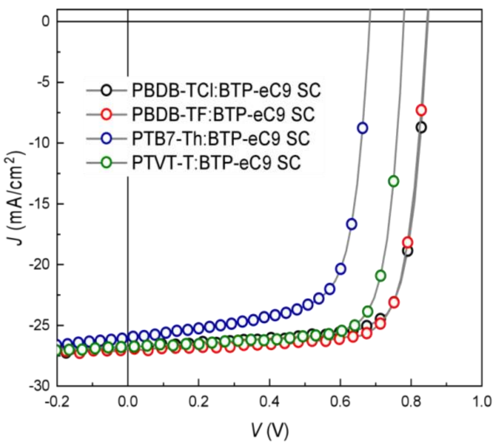

Figure S11 | The current density-voltage *J*-*V* curves of 0.04 cm^2 OSCs with PEDOT:PSS/BHJ/PDINN layers fabricated by spin coating. The testing is carried out under the illumination of AM 1.5G 100 mW/cm^2. The BHJ materials are PBDB-TF:BTP-eC9, PTB7-Th:BTP-eC9 and PTVT-T:BTP-eC9. The ink preparation conditions can be seen in **Materials and Methods** section.

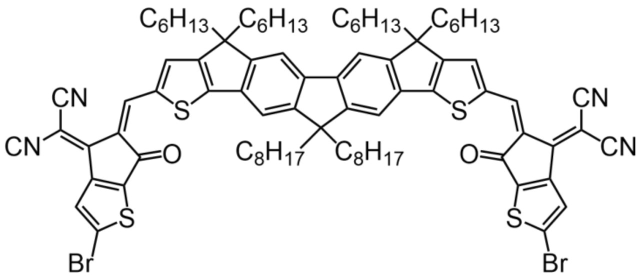

Figure S12 | The molecular structure of FTCC-Br.

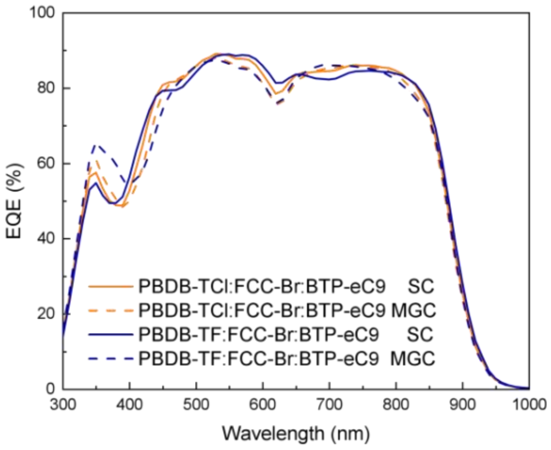

Figure S13 | The EQE spectra of the OSCs based on ternary BHJs. The corresponding *J*-*V* curves of the OSCs based on ternary BHJs have been shown in **Fig. 3l** and **3m**.

Table S3 | The J_{sc} of ternary OSCs obtained from *J*-*V* curves and EQE spectra.

BHJs	J_{sc} calculated from EQE spectra (mA/cm^2)	Deviations from J_{sc} obtained from *J*-*V* curves (mA/cm^2)
PBDB-TCl:FTCC-Br:BTP-eC9 SC	26.53	1.11%
PBDB-TCl:FTCC-Br:BTP-eC9 MGC	26.11	1.43%
PBDB-TF:FTCC-Br:BTP-eC9 SC	26.58	1.23%
PBDB-TF:FTCC-Br:BTP-eC9 MGC	26.15	1.21%

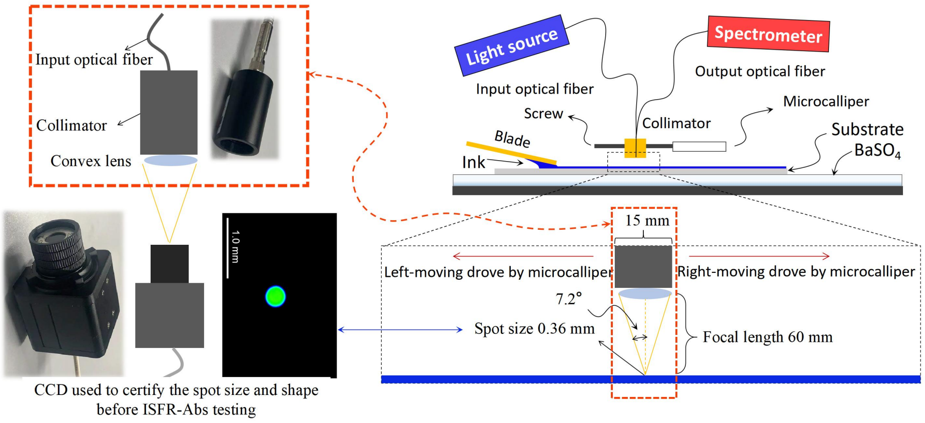

Figure S14 | The instruments of ISFR-Abs experiment.

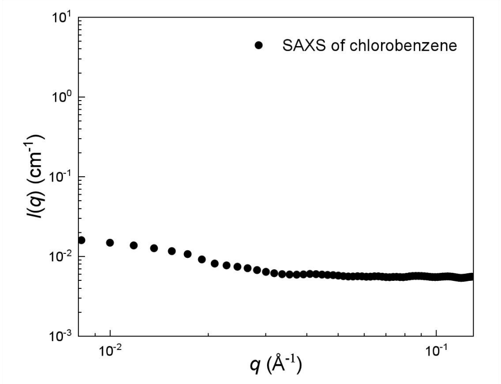

Figure S15 | The SAXS data of solvent (chlorobenzene).
